# Maternal health care access among migrant women labourers in the selected brick kilns of district Faridabad, Haryana: mixed method study on equity and access

**DOI:** 10.1186/s12939-018-0886-x

**Published:** 2018-11-20

**Authors:** Archana Siddaiah, Shashi Kant, Partha Haldar, Sanjay K. Rai, Puneet Misra

**Affiliations:** 1 Department of Community Health, St John’s National Academy of Health Sciences, No 321 Annex 1, Sarjapur road, John Nagar, Kormangala, Bengaluru, 560034 India; 2 Centre for Community Medicine, All India Institute of Medical Sciences, Ansari Nagar, New Delhi, India

**Keywords:** Maternal healthcare, Migrant, Brick kiln, Mixed method study, Equity, Access, India

## Abstract

**Background:**

Socio-economic inequity leads to health inequity. Inequity is closely intertwined with internal migration. This study was planned with the objective of documenting the maternal health care utilization among women labourers working in brick kilns situated in an area of Haryana, north India.

**Methods:**

A community based mixed method study was done in select brick kilns of Faridabad district in north India. A mixed method study was done to assess maternal health care utilization in a sample of 500 women in the reproductive age group. Focus group discussions were also carried out. Descriptive analysis was done. Qualitative data was analysed using the thematic framework approach.

**Results:**

The mean age of the women was 30 (SD 0.3) years. Mean number of pregnancies per woman was 3.1 (SD 1.7). Only 22.9% ever had institutional delivery. About one third of women had ever received cash benefit under Janani SurakshaYojana (JSY) or had ever used free ambulance services. Seven major themes emerged from the qualitative analysis. Important themes include-Gaps in knowledge regarding local health system; Sub-standard private health care delivered at brick kilns prevent migrants from accessing the basic public health services; Misconceptions and mistrust about public health system influenced maternal health care utilization; Barriers to avail universal health coverage: location of brick kilns, time, apathy of public health system, partial health insurance cover.

**Conclusions:**

A typical migrant woman labourer in the brick kiln was an illiterate, had migrated from poor states, belonged to a socially disadvantaged community and worked long hours, and had been doing so for many years. This study has identified migrant women working in brick kilns as a vulnerable population subgroup in terms of maternal health utilization. To achieve universal health care it is important to understand the needs of all population subgroups and make concerted efforts at the health system level.

## Introduction

One of the most pressing global issues is the growing rates of socio-economic inequity [1]. India is the second most populous country in the world with a fast-growing economy which has become more unequal in recent years [2]. Inequity is closely intertwined with internal migration [3]. The National Sample Survey Organization (NSSO) has defined migrants as those for whom the last usual place of residence, where the person had stayed continuously for a period of 6 months or more, is different from the present place of enumeration [4]. According to estimates three out of every ten Indians are migrants [3]. Such migrants mainly work in the informal sector, are responsible for 90% of the workforce and about 50% of the national product [5]. In India, informal sector consists of industries related to construction, agriculture, textiles, brick making, stone quarries and many others [5]. The brick kiln industry is a labour intensive industry involved in producing bricks which are an indispensable component of construction activity. The brick kiln industry in India is next to China in terms of global production having more than 100,000 kilns, employing about 10 million workers [6]. Labourers usually belong to underprivileged sections of society especially rural areas and indulge in circular migration i.e., seasonal migration typically seeking work. This is emerging as a dominant form of migration amongst poorer groups in India [7, 8]. These migrants go with family to work in brick kilns along with their children. This seasonal migration varies from 4 to 6 months in duration usually during September to June which is an agriculturally inactive period in their native villages.

Due to India’s fast urbanization, building construction is estimated to grow at a rate of 6.6% per year between 2005 and 2030 [9]. Naturally, there would be a proportional growth of brick industry and migrants working in this industry. Consequently, the health of the workers in this industry assumes importance. Maternal and child health is largely influenced by social determinants. Circular migration affects health service utilization and thus the health status of women and children who are most vulnerable. Efforts by the government in increasing access to maternal health has been unequal and many such migrant women still lack access to basic maternal health care [10]. Countries like Sri Lanka, Malaysia and Hong Kong which are nearing achieving universal health care are the ones in which their poorest people made no use of private health care [1]. Increasingly, many global studies have found disparities in terms of health among migrants and natives in terms of access to basic health care [11–15].

Even though many studies on migrants are directed towards non-communicable diseases, few studies have reported maternal and child health status of migrants. Surveys such as National Family Health Survey (NFHS), and the District Level Household Survey (DLHS) did not consider migration as a variable affecting the health status in general, and maternal health in particular [16, 17]. Therefore, we wanted to assess the effect of socially produced inequities on maternal health care utilization among migrants which remains unknown. Hence, we planned this study with the objective of documenting the maternal health care utilization among women labourers working in the brick kilns situated in an area of Haryana, north India. Studying this would help us identifying actionable areas which can be aimed to reduce health inequities in such vulnerable settings.

## Methodology

### Study design

This study was an exploratory, community-based, mixed method study [18], having both quantitative (cross sectional) and qualitative components.

### Study setting

Faridabad is a district of Haryana in north India, constituting a part of National Capital Region. According to 2011 census, Faridabad recorded a growth rate of 31.7% compared to 20% for whole of Haryana [19]. Ballabgarh was one of the administrative blocks in Faridabad where two Primary Health Centres (PHCs) namely, Dayalpur and Chhainsa are situated [20]. Study was conducted in the brick kilns that were located within these two PHC areas. There was a total of 70 brick kilns in this area. The total number of labourers varied between brick kilns, approximately 20 to 100 families per brick kiln.

### Study population and study period

For this study migrant women in the reproductive age (15–45 years) working in the brick kilns that were situated in and around the above mentioned PHCs were included. Adult men and women labourers in these brick kilns were included for focus group discussions (FDGs). The study was conducted from April 2015 and Dec 2015.

### Sample size

Since the exact count of migrant women in the reproductive age (15–45 years) working in the brick kilns was not available, an estimation of number of women labourers at brick kilns was done by interviewing all brick kiln supervisors telephonically. The estimated number of such women labourers was 1838. Since this was an exploratory study it was decided to study 500 women. Two stage random sampling was done. Inclusion criteria were women within the reproductive age group, history of at least one child birth, currently working in brick kiln, and able to understand and speak in Hindi.

### Quantitative study tools

An interview schedule was prepared in English and translated to local language (Hindi). The interview schedule broadly captured the participant’s demographic and socio-economic information, report of antenatal care, receipt of iron and folic acid (IFA) supplements, and maternal health care access for the most recent pregnancy. Interview schedule was finalised after pretesting by one of the co-investigators in one brick kiln which was excluded from the present study. Four research assistants were trained over 3 days to collect data. They conducted face to face interviews with all participants in their homes at their respective brick kilns. Co-investigator supervised the data collection in the field.

### Qualitative

Focus groups were considered ideal for exploring the dynamics of migration and health service utilization and also to understand migrants’ perspective on this. An FGD guide was prepared with eight open ended questions with suitable probes. Separate FGDs (6–8 participants per FGD) were conducted for men and women. A total of five FGDs were conducted by AS (doctor, trained in qualitative research) in Hindi in the brick kilns. With the consent of each participant, FGDs were audio taped. Field notes were made, when required. Each FGD lasted for 35–50 min. After each interview, the key points were summarized by the interviewer and verified as a way of participant validation.

### Statistical analysis

Data was entered into Epi Info 7 software, and analysed in Stata12 (Stata Statistical Software TX: StataCorp LP). Logistic regression was done to determine the predictive factors for maternal health care utilization. Lorenz curve and Gini index was also calculated for measuring inequity. Thematic framework approach was used for qualitative data analysis [21, 22]. A descriptive content analysis by manual coding was done by two independent, trained researchers (AS, PH) to generate basic themes (Table 1). Results were organized under several broad themes. Verbatim quotes were reported for each theme. Results were reported as per the consolidated criteria for reporting qualitative studies (COREQ) guidelines [21].

**Table 1:** Thematic framework used for understanding the maternal health care utilization of migrant women working in selected brick kilns in Faridabad, India

1	*Reasons for migration to the place of work*
1.2	Repayment of loan
1.3	Inability to earn livelihood at the place of origin
1.4	Delayed disbursement of NREGA payments
2.1	*Work pattern in brick kilns*
2.2	Labor intense work
2.3	Long working hours
3	*Facilities available at brick kilns*
4	*Public health system at the place of work*
5.1	*Available health care*
5.2	Non-utilization of public health facilities
5.3	Private health system at the place of work
6.1	*Maternal health care availed by laborers*
6.2	Janani Suraksha Yojana
6.3	Knowledge about emergency transport facility
7	*Utilization of social service schemes*
8	*National health insurance coverage (RSBY)*
9.1	*Inability to avail maternal health care*
9.2	Lack of time, and awareness
9.3	Failure of public health system in planning migrant health services
9.4	Private providers more appealing to laborers
10	*Possible ways to deliver health care to migrant laborers in brick kilns*

*NREGA- National Rural Employment Guarantee Scheme; RSBY-Rashtriya Swasthya Bhima Yojana*

## Ethical issues

Ethical clearance was obtained from the Ethics Committee of All India Institute of Medical Sciences, New Delhi before conducting the study (Ref no- RT-8/25.02.2015). Informed written consent was taken from participants and the left thumb print was taken for those who were unable to write. Upon completion of the study, a poster was distributed to all brick kilns which contained details about the nearest government health facilities and details of maternal health services available there.

## Results

### Quantitative

A total of 518 women were interviewed in the quantitative survey. The mean age of the women was 30 (SD 0.3) years and mean age at marriage was 17.3 (SD 2.3) years. Majority of women (67.3%) were illiterate and 36.1% of their spouse was illiterate (Table 2). Almost 60% of women reported possessing Below Poverty Line (BPL) card, and 47.9% had Rashtriya Swasthya BhimaYojana (RSBY- national health insurance) card [23].

**Table 2:** Socio-demographic details of the migrant women labourers working in selected brick kilns in Faridabad, India (*n* = 518)

Sl no	Variables	Number	Percentage
1	*State of origin*		
	Chhattisgarh	363	70.1
	Uttar Pradesh	56	10.8
	Rajasthan	24	4.6
	Others	75	14.5
2	Education status of women (illiterate)	349	67.3
3	Education status of husband (illiterate)	187	36.1
3	Involved in agricultural work in native place	476	91.9
4	*Caste*		
	SC/ST	423	81.7
	Others	95	18.3
5	*Most important reason for working in brick kilns*		
	Repayment of debt	230	44.4
	More money in brick kilns	145	28.0
	No work in native place	131	25.3
6	Mean years of working in brick kilns (SD)	7.4 (3.7)	–
7	Women’s mean hours of working in brick kilns (SD)	10.7 (2.5)	–
8	Median income per season	₹ 30,000	–

*SC/ST- Scheduled caste and Scheduled tribe; SD- Standard deviation*

Mean age at first pregnancy was 18.8 (SD 2.2) years. Only 22.9% ever delivered a baby in a hospital. Availability of good hospital facility encouraged majority of participants (60.1%) to have institutional delivery. The most common reason for home delivery was ease of having home delivery and absence of any problem during pregnancy (64.7%). About one third of women had ever received cash benefit under Janani Suraksha Yojana (JSY) [24] or had ever used free ambulance services (Tables 3 and 4). Majority of participants (67.4%) preferred government facility for maternal health care as they were provided services free of cost (Table 5).

**Table 3:** Details of last pregnancy and delivery of migrant women labourers working in selected brick kilns in Faridabad, India (*n* = 518)

Sl no	Variable	Number	Percentage
1	*Details of last pregnancy*		
1.1	Received some antenatal care during the last pregnancy	389	75.1
1.2	Received iron and folic acid tablet	200	38.6
1.3	Haemoglobin test done	336	64.9
1.4	Received tetanus toxoid injection	416	80.3
2	*Place of delivery*		
2.1	Home delivery	399	77.1
2.2	Hospital delivery	119	22.9
3	*Contraceptive usage*		
3.1	Ever used any contraceptive	145	28.0
3.2	Underwent tubectomy (*n* = 145)	106	73.1
4	*Facilities available for pregnant women*		
4.1	Ever heard about JSY services	233	45.0
4.2	Ever received JSY money	159	30.7
4.3	Ever heard about free ambulance facility	381	73.6
4.4	Ever used free ambulance facility	189	36.5

*JSY Janani Suraksha Yojana*

**Table 4:** Determinants of ANC and place of delivery among migrant women working in selected brick kilns in Faridabad, India (*n* = 518)

Variable	Antenatal care^a^		*p* value	Place of delivery		*p* value
	Adequate (*n* = 278)	Inadequate (*n* = 240)		Institutional (*n* = 175)	Home (*n* = 343)	
*Mean age (SD)*	28.2 (6.3)	32 (6.9)	0.000	26.1 (5.1)	31.9 (6.8)	0.000
*Stay in brick kiln*						
Long	95 (18.3)	130 (25.1)	0.000	58 (11.2)	167 (31.7)	
Short	183 (35.3)	110 (21.2)		117 (22.6)	179 (34.6)	0.000
*Participant Education*						
Illiterate	169 (32.6)	180 (34.8)		92 (17.8)	257 (49.6)	
Literate	109 (21.0)	60 (11.6)	0.001	83 (16.0)	86 (16.6)	0.000
*Occupation*						
Agriculture	238 (45.0)	165 (31.8)		126 (24.3)	102 (19.7)	
Others	40 (7.7)	75 (14.5)	0.000	49 (9.5)	241 (46.5)	0.000
*Income*						
≤25,000	97 (18.7)	112 (21.6)		89 (17.2)	120 (23.2)	
>25,000	181 (34.9)	128 (46.3)	0.006	86 (16.6)	223 (43.1)	0.000
*H visit*						
Yes	225 (43.4)	105 (20.3)		107 (20.7)	223 (43.1)	
No	53 (10.2)	135 (46.3)	0.000	68 (13.1)	120 (23.2)	0.386
*ASHA visit*						
Yes	247 (47.7)	160 (30.9)		139 (26.8)	268 (51.7)	
No	31 (6.0)	80 (15.4)	0.000	36 (7.0)	75 (14.5)	0.734
*Used ambulance*						
Yes	–	–		87 (16.8)	102 (19.7)	
No	–	–	–	88(17.0)	241 (46.5)	0.000

^*a*^ *Adequate Antenatal care was defined as received some antenatal care, TT injections, and IFA tablets*

**Table 5:** Reasons for health system preference for general ailments and maternal health care among migrant women labourers working in select brick kilns in Faridabad, India (*n* = 518)

Preference for general ailments (*n* = 509)^a^	Reason for preference	Number (Percent)	Preference for maternal care (*n* = 505)^b^	Reason for preference	Number (Percent)
Government 204 (39.8)	Accessible	23 (11.3)	Government 340 (67.4)	Accessible	17 (5.0)
	Free treatment	139 (68.1)		Ambulance	54 (15.9)
	Quality care	34 (16.7)		Free treatment	159 (46.8)
	Others	8 (3.9)		Quality care	45 (13.2)
	–			JSY	43 (12.6)
	–			Others	22 (6.5)
Sub-total		204 (100)			340 (100)
Private 305 (59.5)	Government not good	41 (13.4)	Private 164 (32.6)	Accessible	26 (15.9)
	Accessible	84 (27.5)		Doctor came home	8 (4.9)
	Doctor came home	37(12.1)		Quality care	57 (34.8)
	Quality care	126 (41.3)		Others	73 (44.5)
	Others	17 (5.6)		–	
Sub-total		305 (100)			164 (100)

^*a*^ *9 participants reported no preference*

^*b*^ *13 participants reported no preference*

Variables were tested for association with outcome variables viz. ANC and place of delivery. (Table 4). Variables that were significantly associated were put into the regression model. Logistic regression analysis showed that younger age, involved in agricultural work in the place of origin, and visit by health worker or ASHA were some of the important predictors for maternal health care utilization. (Tables 6 & 7).

**Table 6:** Predictors of ANC using logistic regression among migrant women working in selected brick kilns in Faridabad, India (*n* = 518)

Sl no	Predictor variable	Antenatal care^a^		Odds ratio with 95% confidence interval
		Adequate (*n* = 278)	Inadequate (*n* = 240)	
1	*Participant age*			
	17–25 (*n* = 178)	71.35	28.65	1
	26–35 (*n* = 236)	48.73	51.27	0.7 (0.6–1.0)
	36–45 (*n* = 104)	34.62	65.38	2.25 (1.63–3.12)
2	*Participant education*			
	Literate (*n* = 109)	64.50	35.50	1
	Illiterate (*n* = 169)	48.42	51.58	1.07 (.67–1.68)
3	*Stay in brick kilns*			
	Short (*n* = 293)	62.46	37.54	1
	Long (*n* = 225)	42.22	57.78	1.27 (.82–1.97)
4	*Income*			
	> 25,000 (*n* = 309)	58.58	41.42	1
	≤25,000 (*n* = 209)	46.41	53.59	.61 (.40–.92)
5	*Work in native place*			
	Agriculture (403)	59.06	40.94	1
	Non agriculture (*n* = 115)	34.78	65.22	2.17 (1.33–3.53)
6	*Health worker visit*			
	Yes (*n* = 330)	68.18	31.82	1
	No (*n* = 188)	28.19	71.81	4.06 (2.57–6.42)
7	*ASHA worker visit*			
	Yes (*n* = 407)	60.69	39.31	1
	No (*n* = 111)	27.93	72.07	1.96 (1.12–3.41)

^*a*^ *Adequate Antenatal care was defined as received some antenatal care, TT injections, and IFA tablets*

**Table 7:** Predictors of place of delivery using logistic regression among migrant women working in selected brick kilns in Faridabad, India (*n* = 518)

Sl no	Predictor variable	Place of delivery		Odds ration with 95% confidence interval
		Institutional (*n* = 175)	Home (*n* = 343)	
1	*Participant age*			
	17–25 (*n* = 178)	57.30	42.70	1
	26–35 (*n* = 236)	27.54	72.46	.9 (.6–1.2)
	36–45 (*n* = 104)	7.69	92.31	2.88 (2.03–4.08)
2	*Participant education*			1.64 (1.03–2.59)
	Literate (*n* = 109)	49.11	50.89	1
	Illiterate (*n* = 169)	26.36	73.64	1.60 (1.0–2.5)
3	*ANC utilization* ^*a*^			
	Adequate (*n* = 278)	41.01	58.99	1
	Inadequate (*n* = 240)	25.42	74.58	1.5 (.9–2.4)
4	*Income*			
	> 25,000 (*n* = 309)	27.83	72.17	1
	≤25,000 (*n* = 209)	42.58	57.42	2.3 (1.5–3.7)
5	*Work in native place*			
	Agriculture (403)	31.27	68.73	1
	Non agriculture (*n* = 115)	42.61	57.39	.34(.2–.5)
6	*Used ambulance*			
	Yes (*n* = 189)	46.03	53.97	1
	No (*n* = 329)	26.75	73.25	2.3 (1.4–3.8)

^*a*^ *Adequate Antenatal care was defined as received some antenatal care, TT injections, and IFA tablets*

**Fig. 1:**
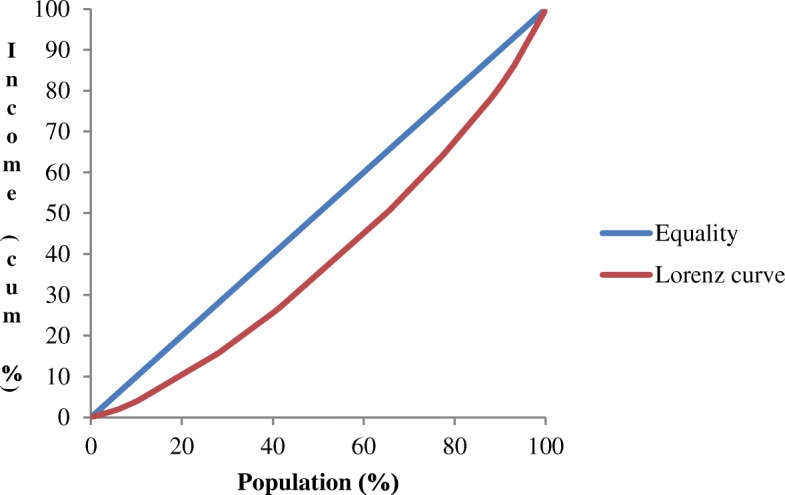
Lorenz curve for household income inequality among the migrant women labourers working in select brick kilns in Faridabad, India (*n* = 518)

The Gini index was found to be 0.2184. Since the inequality is more if this index is closer to 1, the inequality in our sample was not very high. Figure 1 depicts the Lorenz curve using the population proportion against the cumulative percentage of income.

### Qualitative

Five FGDs were conducted, three involving all women labourers and two involving men labourers. All of them aged between 18 and 46 years of age. Seven major themes emerged from the analysis, i.e., 1) Inability to earn livelihood at place of origin; 2) Laborious work in brick kilns often involving pregnant women; 3) Gaps in knowledge regarding local health system; 4) Sub-standard private health care delivered at brick kilns prevent migrants from accessing the basic public health services; 5) Misconceptions and mistrust about public health system influenced maternal health care utilization; 6) Barriers to avail universal health coverage: location of brick kilns, time, apathy of public health system, partial health insurance cover;7) As part of universal health coverage concerted efforts by the public health system to address maternal health needs of migrant women (Table 8).

**Table 8:** A brief description of the framework used in qualitative data analysis understanding the maternal health care utilization of migrant women working in selected brick kilns in Faridabad, India

*Thematic framework components and quotes*	*Codes*	*Summary*	*Categories*	*Subthemes*	*Themes* ^*a*^
*Reasons for migration to the place of work* “NREGA payment is made after 2 ½ months...there is no timely payment of wages [at the place of origin]” “There is no work at my native place”	Employment issues at the place of origin	Failure of government to make timely NREGA payments along with lack of provision generating income compelled migrants to seek work elsewhere	Difficulty in earning livelihood at the place of origin	Delayed disbursement of NREGA	1
*Labour intense work in brick kilns* “We usually work from 4 in the morning till 7 in evening…once in a fortnight we get 2–3 days off” “Work here is strenuous”	Working condition of migrant laborers working in brick kilns	Many laborers including pregnant women worked for a long time in the brick kilns and also availed few number of leaves as obliged by their employer	Difficult working conditions prevalent in the brick kilns	Long working hours involving strenuous work.	2
*Public health system at the place of work* “Pregnant females are unaware about health matters” “People have told us that doctor comes here [brick kilns] to check pregnant females”	Presence of public health facilities	Due to faulty perceptions, lack of awareness migrant laborers rarely availed health services from the public health facilities	Unawareness about local public health facilities	Local public health system less utilized	3
*Private health system at the place of work* “We usually take medicines from one RMP^a^ doctors who come in bicycle [to brick kiln]…and give their mobile numbers…when in need we will call them…for fever and cough we will have to spend ₹100 and for fluid infusion ₹1000..”	Private health care easily available at the place of work	Failure of public health system to identify and step up in providing health services, makes unqualified practitioners more appealing to the laborers, who usually provide substandard but costly health services.	Private health care delivered at the place of work	Substandard but costly health care availed from private providers Visiting brick kilns private providers induce migrants labourers to utilize private health service	4
*Maternal health care availed by laborers* “Sometimes we send pregnant women back home [place of origin] for delivery…in case of emergency we call our supervisor to take patient to government hospital” “We know of a RMP here and a hospital in Chhattisgarh. We have no information about ambulance.”	Health care during pregnancy and child birth	For child birth public health facility was preferred. However, many sent pregnant women to their place of origin for child birth resulting in discontinuation of health services. Lack of information about ambulance facilities at the place of work.	Discontinuation in availing maternal health care	Pregnant women working in brick kilns sent home for child birth	5
*Perceived inability to avail maternal health care* “There is no time left [to visit health facilities]” “We don’t know what kind of services are available here for pregnant women. Even if someone becomes ill we don’t know what to do”	Barriers for universal health coverage	Long working hours perceived to be barrier to visit nearby health facilities. Also, geographical location of brick kilns restricted laborers from visiting health centers after work hours.	Poor utilization of health services	Lack of time in brick kilns for visiting health facilities	6
*Possible ways to deliver health care to migrant laborers in brick kilns* “Doctors should visit bhatta [brick kiln] in evening” “It [visit to brick kilns by health providers] is good time in afternoon for health services”	Concerted efforts required	Need for health providers from the public health system for providing primary care in brick kilns was strongly desired.	Migrant population need to be covered under public health system	Need for providing primary health care in brick kilns Outreach services in brick kilns	7

^*a*^ *Themes 1-Inability to earn livelihood at place of origin hence working in brick kilns was a feasible option; 2-Laborious work in brick kilns often involving pregnant women; 3-Gaps in knowledge regarding local health system; 4-Substandard informal health care delivered at brick kilns prevent migrants from accessing the basic public health services; 5-Misconceptions and mistrust about public health system influence maternal health care utilization; 6-Barriers to avail universal health coverage: location of brick kilns, time, apathy of public health system, partial health insurance cover;7-As part of universal health coverage concerted efforts by the public health system to address maternal health needs of migrant women*

*NREGA National Rural Employment Guarantee Scheme; RMP Registered Medical Practitioner. However, in this context informal health provider without any government recognised medical degree*

Gaps in knowledge regarding local health system: Many participants were not aware of the existing local health systems which impaired their healthcare utilization. Many also mentioned that they knew about free ambulance facility at their place of origin but not at the place of work.

*Male participant 4: “We have no information about ambulance”.*

By visiting brick kilns private providers induce migrant labourers to utilize private health service: Poor access to public health systems facilitated private providers to target such migrant labourers for monetary gains.

*Male participant 2: “We usually take medicines from one RMP doctor* [private provider] *who come in bicycle* [to brick kiln]*…”.*

*Female participant 8: “RMP charges rupees 150 for medicines and rupees 500 for injections”.*

Pregnant women working in brick kilns sent home for child birth: For child birth public health facility was preferred. Due to various reasons women labourers were unable to avail maternal health services at the place of work.

*Female participant 10: “Sometimes we send pregnant women back home* [place of origin] *for delivery…in case of emergency we call our supervisor to take patient to government hospital”.*

Failure of public health system to provide health services for migrants: Apathy of public health system to act to the needs of migrants emerged as one of the important themes.

*Female participant 12: “We don’t know what kinds of services are available here for pregnant women. Even if someone becomes ill we don’t know what to do”.*

Summary of results of qualitative analysis is summarised as barriers and possible solutions for improving maternal health care utilization for migrant women labourers (Table 9).

**Table 9:** Barriers and solutions identified for maternal health care utilization by migrant women working in selected brick kilns in Faridabad, India

Barriers	Solutions
Lack of income generating activities in the place of origin resulting in migration	Rural employment Government must step up efforts such as NREGA to improve the livelihood of people; early payment of NREGA wages;
Low awareness	IEC about the maternal health issues
Prolonged working hours in the brick kilns	Prescribing minimum work time for the labourers working in unorganised sector
Unfamiliarity of local setting in the place of work	Assistance from the brick kiln employers; IEC
Private providers capitalizing on the prevailing situation of migrant labourers preventing them from seeking basic public health care	Sensitizing private providers about the need for migrant labourers to have access for universal health coverage; Regulation of unqualified private providers;
Absence of an existing channel thorough which public health care can be delivered to migrant labourers; Disruption in continuing maternal health care at the place of work	Deployment of health providers such as ASHA; Migrant mobile health unit to help migrant labourers continue accessing public health system even at the place of work
Underutilization of emergency transport facility, JSY	Awareness campaign regarding birth preparedness and complication readiness
Issues in availing benefits from the national insurance scheme	Strengthening of RSBY to achieve universal health coverage
Public health system’s apathy in providing migrant specific health care	Strategies targeting migrant labourers to be incorporated in the National health programmes such as NHM

*NREGA- National Rural Employment Guarantee Scheme; IEC-Information Education and Communication; ASHA-Accredited Social Health Activist; JSY-Janani Suraksha Yojana; RSBY-Rashtriya Swasthya Bhima Yojana; NHM-National Health Mission*

## Discussion

This study was done to assess the maternal health care utilization among migrant women. The mean age of women was 30 (SD 0.3) years and the mean age at first pregnancy was 17.3 (SD 2.3) years. Previous studies have reported lower age at first pregnancy among migrants compared to natives [25]. Many women labourers in brick kilns matched this socio-demographic profile.

### Inadequate antenatal care

WHO recommends that pregnant women should receive focussed antenatal care as it is an important determinant of safe delivery [26]. Our study found inadequate antenatal care. In this study although 3/4th of all women had at least one contact with formal health sector yet only 38.6% had received iron folic acid tablet. In rural Haryana 18.6% pregnant women consumed 100 iron and folic acid (IFA) tablets during pregnancy [16]. Given the low receipt and still lower compliance of IFA tablets, the problem of anaemia remains largely unaddressed among most of the participants. Poor access to basic health care due to socioeconomic disparities is associated with worse healthcare utilization and health outcomes [27–29]. Women in brick kilns were living in a medically underserved region and faced barriers to health care access such as inaccessible transportation. There was no special effort from public health system to reach out to migrants. In such a situation migrants relied on unregulated private providers for basic health care.

### Place of delivery

This study was conducted 10 years after Government of India (GOI) launched National Health Mission (NHM), still only 22.9% had institutional delivery while the institutional delivery rate for rural Haryana that was 80%.The frequently mentioned reasons for home delivery in this study was “no problem during pregnancy”, and “home delivery was easy”. Many women were not aware of the local health facilities. So, it is reasonable to assume that birth preparedness was lacking. Majority (81.7%) of women in our study belonged to SC/ST, a disadvantaged section which is associated with poor uptake of antenatal care. Age wise break up of institutional delivery showed that older women had less institutional delivery rate. Women’s social class, and more number of years in brick kilns along with location of brick kilns might have limited the access to institutional care at the time of delivery. Circular migration has also shown to affect health service uptake at place of origin [7]. Many preferred government health facility for care during pregnancy and opted for institutional delivery if good facility, free treatment, and quality of care was available. Unfortunately, this was not the case at the place of work which explains the low proportion of institutional delivery.

### Referral transport, JSY, and health insurance

Even though two thirds of women were aware about free referral transport, only 36.5% had ever utilised it. A time series analysis done in 2013observed that institutional deliveries in Haryana rose significantly after the introduction of free referral transport [30]. Rural women utilized ambulance for reaching a health facility during labour irrespective of presence or absence of complication. However, participants in this study revealed that they did not utilise ambulance during labour as they felt they did not have any problem. Many also mentioned that they knew about free ambulance facility at their place of origin but not at their work place. Some participants mentioned that they would go back to their place of origin before delivery making continuity of care difficult. However, it is known that women would be more comfortable at their place of origin. There may be many reasons for this such as social support, familiarity with the health facility. Interestingly, only 45% of participants had heard about JSY scheme, which was launched as a flagship programme under NHM in 2005 to increase institutional delivery. GOI introduced the RSBY in 2012, a national insurance scheme for workers of unorganised sector [23]. In our study partial health insurance cover: barrier to avail universal health coverage was an important theme. This was also affirmed during quantitative analysis as less than half possessed an RSBY card. Poverty, illiteracy, residing away from native place, location of brick kilns, and frequent movement contributed to less utilization of such schemes among migrants which in turn leads to unequal access to health care.

### Possible solutions

Firstly, mapping of such unorganised migrant settlements and reaching out to them through the public health system is needed. Intense information education and communication activities targeted to migrant populations about the existing public health facilities, free referral transport, RSBY is an important strategy. Since JSY is a 100% centrally funded scheme, the health system could become flexible to provide cash incentives for institutional delivery to migrants at their place of work. It must be ensured that migrants avail maternal and child health care with zero out-of-pocket expenditure irrespective of work place. This is expected to increase the uptake of maternal health services. Under the mobile health map programme, migrant populations are accessing primary health care in the United States [31, 32]. Similarly, considering the seasonality of migration, local public health system must be sensitised to provide outreach maternal health services using dedicated migrant mobile health units involving frontline workforce such as ASHA (Accredited Social Health Activist). There is a scope to report how social inequity influence health indicators in national level surveys. For this NFHS and DLHS must include such migrant population in their surveys. Concerted efforts by the concerned stake holders within and outside public health system to address maternal health needs of migrant women is need of the hour to achieve universal health coverage.

### Strengths and limitations

The strength of our study was its mixed methods design with the quantitative and qualitative components complementing each other. Limitations include lack of information on each pregnancy and its complications, and outcomes. Under reporting of heath care access could be possible since it was self reported. Cross sectional nature of the study did not allow us to capture the information over a longer time frame which would have given more insight for comparing maternal health care access at the place of origin and place of work. Since this was an exploratory study, results of the statistical tests needs to be interpreted cautiously. However, we feel that this study has provided some of the important predictors of low maternal health care utilization which needs to be confirmed by robust epidemiological studies. Also, only labourers from brick kilns (a part of unorganised sector) were included in this study.

## Conclusions

Our study revealed that a typical migrant woman labourer in brick kiln was an illiterate; primarily worked as an agriculture labourer at her place of origin; had migrated from poor states; she belonged to the socially disadvantaged community and had chosen the present occupation out of indebtedness; she usually worked long hours and had been doing so for many years. This study has identified migrant women working in brick kilns as a vulnerable population subgroup in terms of maternal healthcare utilization. To achieve universal health coverage, it is important to consider the needs of all population subgroups. Research and advocacy focusing on the health of women migrants in unorganised sector should be encouraged. Unless government provides easily accessible, affordable and equitable health care to all its citizens especially poor migrants in line with natives, it is impossible to reduce the existing health inequity.

## Abbreviations

ASHA: Accredited Social Health Activist
DLHS: District Level Household Survey
FGD: Focus Group Discussion
GOI: Government of India
IFA: Iron and Folic Acid
JSY: Janani Suraksha Yojana
NFHS: National Family Health Survey
NHM: National Health Mission
NREGA: National Rural Employment Guarantee Scheme
NSSO: National Sample Survey Organization
SC/ST: Scheduled Caste and Scheduled Tribe
SD: Standard Deviation
WHO: World Health Organization
